# Identification and analyses of exonic and copy number variants in spastic paraplegia

**DOI:** 10.1038/s41598-024-64922-8

**Published:** 2024-06-21

**Authors:** Anum Shafique, Ayesha Nadeem, Faiza Aslam, Humera Manzoor, Muhammad Noman, Elizabeth Wohler, P. Dane Witmer, Nara Sobreira, Sadaf Naz

**Affiliations:** 1 School of Biological Sciences, University of the Punjab, Quaid-e-Azam Campus, Lahore, 54590 Pakistan; 2 McKusick-Nathans Department of Genetic Medicine, Baylor Hopkins Center for Mendelian Genomics, Baltimore, MD USA; 3 Present Address: Department of Biochemistry, Faisalabad Medical University, Faisalabad, Pakistan

**Keywords:** AP4B1, DDHD2, Exome, Pakistan, Spasticity, SPG11, Genetics, Molecular biology

## Abstract

Hereditary spastic paraplegias are a diverse group of degenerative disorders that are clinically categorized as isolated; with involvement of lower limb spasticity, or symptomatic, where spastic paraplegia is complicated by further neurological features. We sought to identify the underlying genetic causes of these disorders in the participating patients. Three consanguineous families with multiple affected members were identified by visiting special schools in the Punjab Province. DNA was extracted from blood samples of the participants. Exome sequencing was performed for selected patients from the three families, and the data were filtered to identify rare homozygous variants. ExomeDepth was used for the delineation of the copy number variants. All patients had varying degrees of intellectual disabilities, poor speech development, spasticity, a wide-based gait or an inability to walk and hypertonia. In family RDHR07, a homozygous deletion involving multiple exons and introns of *SPG11* (NC000015.9:g.44894055_449028del) was found and correlated with the phenotype of the patients who had spasticity and other complex movement disorders, but not those who exhibited ataxic or indeterminate symptoms as well. In families ANMD03 and RDFA06, a nonsense variant, c.985C > T;(p.Arg329Ter) in *DDHD2* and a frameshift insertion‒deletion variant of *AP4B1*, c.965-967delACTinsC;p.(Tyr322SerfsTer14), were identified which were homozygous in the patients while the obligate carriers in the respective pedigrees were heterozygous. All variants were ultra-rare with none, or very few carriers identified in the public databases. The three loss of function variants are likely to cause nonsense-mediated decay of the respective transcripts. Our research adds to the genetic variability associated with the *SPG11* and *AP4B1* variants and emphasizes the genetic heterogeneity of hereditary spastic paraplegia.

## Introduction

Hereditary spastic paraplegia (HSP) comprise a genetically diverse group of inherited neurologic disorders that share symptoms of progressive lower and upper limb spasticity and muscle weakness resulting from axonal degeneration of the corticospinal tracts^1^. Patients having uncomplicated or pure HSPs may exhibit additional disease features including urinary bladder dysfunction^2^. Complicated or complex HSPs disorders involve varying co-presentations of intellectual disabilities, ataxia, dystonia, seizures, Parkinsonism, neuropathies and visual abnormalities^3,4^. More than 80 inherited types of HSP have been reported, in which autosomal dominant, autosomal recessive, X-linked and maternally inherited patterns are observed^5^. Hereditary spastic paraplegia has a prevalence of 0.1–9.6 in every 100,000 individuals, depending on the geographical location. Autosomal dominant forms have been globally described and are predominantly observed as pure HSP in the majority of the North American and North European hereditary spastic paraplegia cases. Variants of genes commonly described to play roles in these HSP forms are *SPG4*/SPAST, *SPG3A*/ATL1, *SPG31*/REEP1, and *SPG10*/KIF5A^6,7^. On the other hand, autosomal recessive (AR) HSPs are usually more severe and complicated. Among these, *SPG11* and *SPG15* are significant contributors to the recessively inherited HSPs in the Middle East and Northern Africa^8^.

Variants of *SPG11* (OMIM:604360), *PGN* (OMIM:607259), *FA2H* (OMIM:612319), *ZFYVE26* (OMIM:270700), *AP5Z1* (OMIM:613647), *CYP7B1* (OMIM:270800) and *ATP13A2* (OMIM:617225) are mostly associated with the progression of complicated types of recessive hereditary spastic paraplegia^3^. Global contribution of all genes to complicated HSP is not known, though some have been correlated with the phenotypes more often than the others^7,8^. These genes encode proteins that participate in neural growth, cargo trafficking, destruction of misfolded proteins, synthesis of cholesterol and myelin synthesis pathways.

Spastic paraplegia type 11 (*SPG11*) variants have been commonly described as cause of autosomal recessive hereditary spastic paraplegia (ARHSP) in some studies^9^. A thin corpus callosum is observed in 41–77% of reported cases due to *SPG11* variants^10,11^. The *SPG11*-encoded transmembrane protein spatacsin plays a role in intracellular trafficking. Another complex form of HSP known as *SPG54* is caused by recessively inherited variants in *DDHD2*, which encodes the DDHD domain-containing protein. This gene is part of one of the three intracellular phospholipase A1 (iPLA1) families of proteins (DDHD1, DDHD2 and SEC23IP) that are involved in organelle biogenesis and membrane trafficking between the endoplasmic reticulum and the Golgi body.

There are also reports of the involvement of subunits of the AP-4 complex, a heterotetrameric protein, in the pathogenesis of HSP termed *SPG47*. The AP-4 complex is known to play a role in the polarized sorting of cargo in the epithelial cells and neurons. Biallelic variants of genes encoding all the subunits of the AP-4 complex cause HSP. The disease conditions associated with variants of the AP-4 complex are collectively referred to as AP-4 deficiency syndromes^12^. The involvement of all four subunits of the AP-4 complex in the etiology of neurological disorders is evidence for the essential role of this complex in the function of neurons and the brain.

Evaluation of a consanguineous family with multiple patients affected by a recessively inherited disorder is highly successful in identifying the causative variant. The aim of the present study was to molecularly characterize three families, each having two or more HSP patients from the Punjab province in Pakistan. We hypothesized that the obtained information will broaden the genetic spectrum of the disorder. A homozygous deletion encompassing multiple exons and introns of *SPG11*, a homozygous nonsense variant in *DDHD2* and a homozygous frameshift insertion‒deletion variant of *AP4B1* were correlated with the phenotypes of patients in the three families.

## Materials and methods

### Ethical review

The Institutional Review Board of the School of Biological Sciences, University of the Punjab, Lahore, Pakistan (IRB No. 00005281, FWA 00010252), approved the study. Written informed consents were obtained from all the participants. Parents provided consent for their minor children.

### Family recruitment and sample collection

**Figure 1:**
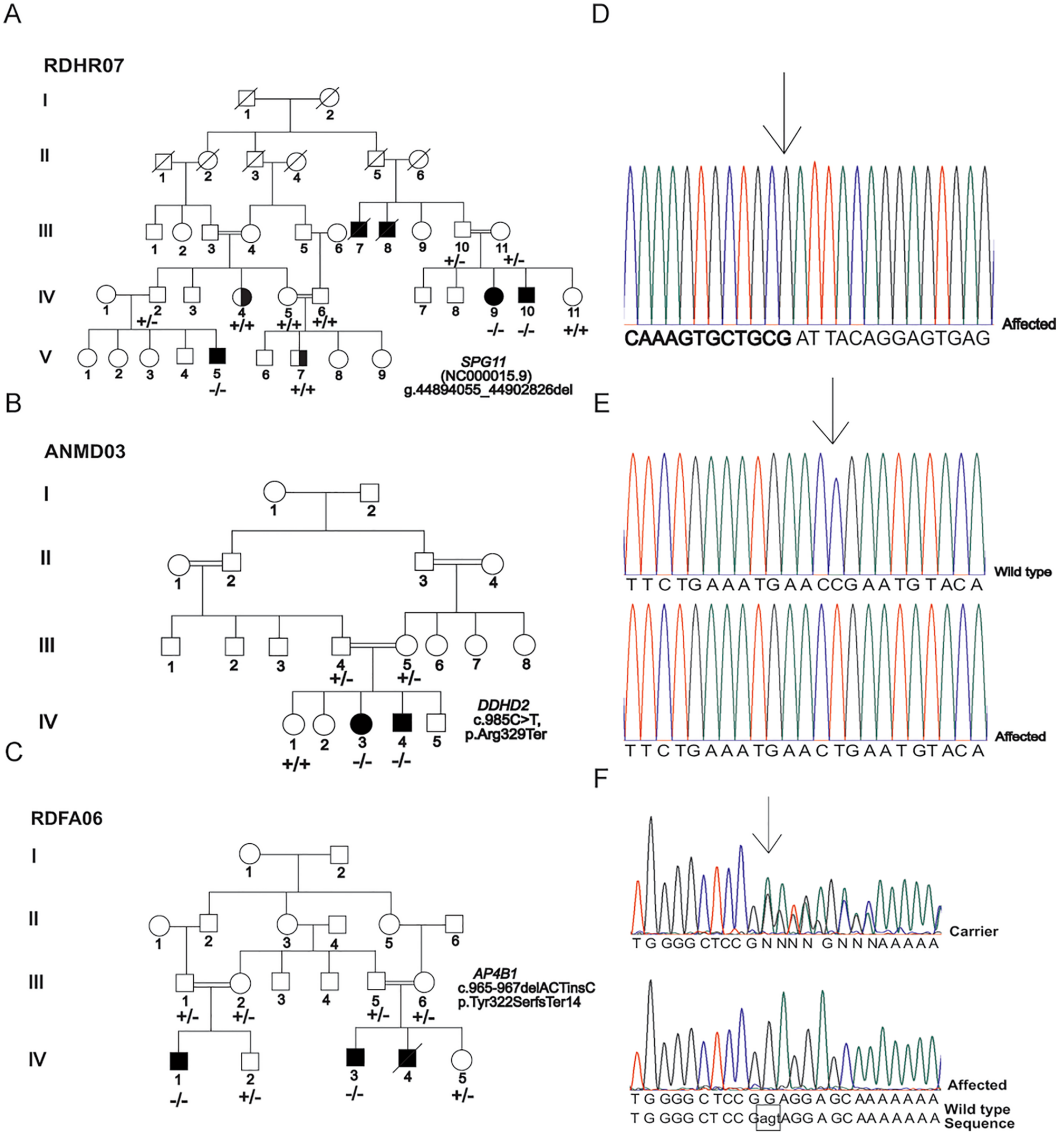
Pedigrees and partial electropherograms. (**A**) Pedigree of family RDHR07. Patients were from multiple generations of the family. Filled black symbols denote those individuals with spastic paraplegia while the half filled symbols indicate those with a spastic /ataxic/dystonic disorder which is different from that of the other patients. The *SPG11* allele genotypes of the participants are indicated under their symbols (+ = wild-type allele, − = deletion allele). (**B**) Pedigree of family ANMD03. Individuals who participated in the study are indicated. The *DDHD2* genotypes are shown below the symbols for individual participants. (**C**) Pedigree of family RDFA06, with individuals affected in two branches. The *AP4B1* genotypes of all the participating individuals are mentioned. (**D**) Partial electropherogram from BTSeq sequencing results of the *SPG11* deletion allele NC000015.9:g.44894055_44902826del. The reverse complement sequence shows intron 20 (at left and base pairs written in bold) continuous with the sequence within intron 18 (at right and base pairs are not written in bold). The arrow indicates the start of intron 20, reading from right to left. Sequence is reverse complement with respect to the cDNA. (**E**) Partial electropherograms of *DDHD2.* Traces from healthy individuals with a wild-type sequence and patients with the variant c.985C > T are shown. The arrow indicates the changed nucleotide. (**F**) Electropherogram of *AP4B1* showing the sequences of the carrier and the affected individuals. The start of the variant c.965-967delACTinsC is indicated by an arrow above the first of the changed nucleotides. The wild-type genomic sequence is shown under the trace of the affected individual, and the deleted/inserted nucleotides are written in lowercase letters and boxed. Sequence is reverse complement with respect to the cDNA.

Families with inherited autosomal recessive forms of movement disorders were identified via personal contacts in Faisalabad and by visiting special education schools in Lahore. Criteria for the recruitment of the families included consanguinity and the presence of multiple individuals in each family with symptoms of spastic movement disorders. Patients in family RDHR07 were investigated first^13^, while those in the ANMD03 and RDFA06 families were newly recruited. A total of seven affected individuals were reported in family RDHR07 (Fig. 1A), five of whom were alive and presented movement disorders with intrafamilial differences. There were two patients each in families ANMD03 (Fig. 1B) and RDFA06 (Fig. 1C). The participants of the three families were videotaped by focusing on the upper and the lower limbs to record unusual movements. The sitting and standing positions were also videotaped. Voice, gait, neck position, eye blinking, and voluntary hand and foot movements were noted. The videos of the patients in family RDHR07 were analyzed previously^13^. Patients from families ANMD03 and RDFA06 were diagnosed by doctors at a local hospital. MRI analyses could not be performed for the patients in the three families. Blood samples were collected from all available family members in the three pedigrees. Genomic DNA was extracted using sucrose lysis and salting out from whole-blood samples.

### Analyses of exome data

Exome sequencing was first performed (Atlas Biolabs, Germany) on samples from individuals III:10, III:11, IV:4, IV:9 and IV:10 for family RDHR07^13^. However, the data analyses revealed no likely pathogenic variants. (This data is no longer available for re-analyses). Exome sequencing was subsequently conducted on the DNA sample from patient IV:10 using the NimblegenSeqCap EZ Human Exome Library v2.0, and the library was sequenced at 100X coverage on an Illumina HiSeq 2000 (Baylor-Hopkins Center for Mendelian Genomics [BHCMG]). The former also completed exome sequencing on DNA of individual IV:3 from family RDFA06. Sample from patient IV:3 of family ANMD03 was analyzed via exome sequencing at Macrogen, Inc., Seoul, South Korea.

The VCF files obtained after exome sequencing were uploaded to the Franklin program (https://franklin.genoox.com) for annotation. The data were filtered to prioritize homozygous variants with control population frequencies less than 1% in the public databases such as gnomADv2, 1000 Genomes and the Exome Variant Server. All exonic and splice site variants (up to the ± 10 position) were examined. Compound heterozygous variants were also subsequently considered. The pathogenicity of each variant was assessed by various online programs, including REVEL, CADD, SIFT and PolyPhen2^14–17^. The evolutionary conservation of amino acids affected by the missense variants was evaluated by observing alignments from 100 vertebrate species (https://genome-euro.ucsc.edu/cgi-bin/hgGateway).

### Copy number variant analysis of exome data

The BAM/BED files of family RDHR07 patient were examined by using ExomeDepth (https://cran.r-project.org/web/packages/ExomeDepth/index.html) for the identification of large genomic deletions and insertions. The data of the affected individual were compared with those from the aggregated reference set^18^. Integrative Genomic Viewer (IGV) was used to display the region of deletion by uploading the BAM files of the patient and that from a control unaffected individual.

### Homozygosity analyses of the exome data

The regions of homozygosity (ROH) were detected for the three participants for whom the exome data were available. The VCF data of the patients from each family were uploaded to Automap (https://automap.iob.ch/) and AgileVCFMapper (http://www.dna-leeds.co.uk/agile/AgileVCFMapper/) programs separately. The default settings were used for each program. The data were visually represented together for all chromosomes by Automap while the individual chromosomes were visualized by AgileVCFMapper. All ROH detected from the exome data spanning at least 1 Mb of homozygous calls were included in the Automap data as well as the number of homozygous variants in these regions. The numbers of total heterozygous and homozygous variants were obtained from the VCF data. The percentages of variants falling in ROH were calculated by dividing the number of homozygous variants observed in ROH regions by that of the total observed variants.

### Primer design, PCR and sequencing

*SPG11* was assessed in all members of the family RDHR07 by designing primers using Primer 3 (https://bioinfo.ut.ee/primer3-0.4.0/). The primers used to detect the wild-type allele spanned exon 20 of NM_025137.4, and *SPG11* at chr15:44,898,070–44,898,552, GRCh37/hg19 (Supplementary Table 1) to amplify a 483 bp fragment. The mutant deletion allele would not be amplified with the abovementioned primers since the corresponding region was expected to be absent from the patients’ DNA. To identify the presence of the variant in all participants and to determine the sequence of the region affected by the deletion, primers located within NM_025137.4, *SPG11* exon 18 and exon 21 (Supplementary Table 1) were designed; these primers would not yield a product from the wild-type allele due to its large size (10.44 kb), while that from the deleted allele was expected to be ~ 1.6 kb.

The ~ 1.6 kb PCR-amplified product from the *SPG11* mutant allele was purified and sequenced by proprietary massively parallel sequencing-based BTSeq technology (Celemics, Seoul, South Korea). This involved fragmenting the PCR products to prepare bar-coded libraries for massively parallel sequencing. The data obtained as fastq files were assembled into contigs and provided as text and trace files for subsequent analyses via standard methods. Thus, the sequence of the ~ 1.6 kb product was obtained as one continuous stretch, which was visualized with SeqMan (DNASTAR, Lasergene, Inc.). BLAST-like Alignment Tool (BLAT) analysis was performed with the obtained nucleotide sequence in the UCSC genome browser GRCh37/hg19 (https://genome-euro.ucsc.edu/cgi-bin/hgGateway). The amplicon sequence that was aligned with the genomic sequence, was marked as present. The unpaired genomic sequence between the alignments was marked as deleted.

The desired DNA sequences of the regions containing selected variants of families ANMD03 and RDFA06 were retrieved from the UCSC Genome browser for primer design (Supplementary Table 1). PCR amplifications were performed for all participants, and segregation analyses were completed by BTSeq technology (for family ANMD03) or by Sanger sequencing using Big Dye Terminators v3.1 (for family RDFA06).

## Results

### Clinical findings

At the time of recruitment, all patients in family RDHR07 were hypothesized to have the same genetic cause of their phenotypes due to their common ancestry (Fig. 1), despite slightly different clinical presentations of the five affected individuals (Table 1). The symptoms indicated complex movement disorders/dystonia/hereditary spastic paraplegia (HSP) with an onset of disease beginning at the age of 3–6 years. Patients IV:9 and IV:10 exhibited severe spasticity of all extremities and could walk only with support and a wide-gait. Other symptoms included dystonic posturing, hypomimia, spontaneous clonus associated with tremors and impaired positional sense. The instinctive Babinski sign on the right was observed for patient IV:9, and the Babinski sign was positive bilaterally for patient IV:10. Patient V:5 had generalized dystonia, including lower face dystonia with dystonic grimacing, cervical dystonia, dystonic postures of the upper and lower extremities and a dystonic gait. Patients IV:4 and V:7 however presented additional clinical manifestations (Table 1). Evaluation of patient IV:4 showed a wide-based spastic-ataxic gait along with a partial decline in the motor function of the right arm, truncal ataxia while sitting, leg pronounced spasticity and proximal weakness. Patient V:7 had an undetermined dystonic phenotype associated with moderate lower limb weakness, right foot inversion and left leg muscle atrophy.

**Table 1:** Clinical Features of the patients from the three participating families.

Feature	Family RDHR07	Family ANMD03	Family RDFA06
Patients	*IV:4* IV:9 IV:10 V:5 *V:7*	IV:3 IV:4	IV:1 IV:3
Variant	Homozygous *SPG11* Intragenic deletion, NC000015.9:g.44894055_44902826del (chr15:44894055-44902826--GRCh37/hg19) (but not in *IV:4* and *V:7*)	Homozygous *DDHD2* c.985C > T p.(Arg329Ter)	Homozygous* AP4B1* c.965-967delACTinsC: p.(Tyr322SerfsTer14)
Age at onset	3–6 years	2 years	1 year
ID	Very mild	Severe	Severe
Cognition	Average	Poor learning	Learning problems and poor responsiveness
Behavior	Normal	Aggressive	Behavior was difficult to assess due to the Intellectual disability
Speech	Dysarthria	Delayed speech	Poor
Seizures	Absent	Present	Absent
Movement	Moderate delay in movements, mixed movement phenotypes of the patients	Severe delay in movements and poor ambulation	Severely delayed and poor movements
Spasticity	Severe presentation in all patients except for individual *V:7*	Present (moderate)	Present (moderate)
Spastic paraplegia	Complicated (IV:9 IV:10 V:5)	Complicated	Complicated
Dystonic Signs	Present in all patients, except *IV:4* and the most severe dystonic signs were observed in patient V:5	Absent	Absent
Hypertonia	Severe	Present	Present in individual IV:3
Hypotonia	Not observed	Not observed	Not observed
Babinski sign	Present in individual IV:9 and IV:10	Absent	Absent
Contractures of the hands and feet	Present	Present	Present
Limb weakness	Mild	Severe	Mild
Gait	The individuals had wide-based spastic- gaits (ataxic in patient *IV:4* and dystonic in patient V:5)	Wide-based gaits	Not able to walk
Peripheral neuropathies	Not observed	Not observed	Not observed
Bladder Issues	Not reported	Not reported	Not reported
Parkinsonism features	No features observed	No features observed	No features observed

ID, Intellectual disability, Patients ID shown in italic represent those in family RDHR07 who shared most features with others in their family except that *IV:4* had ataxia while *V:7* had dystonia and undetermined weakness. These two did not have *SPG11* related spastic paraplegia.

In family ANMD03, the two patients had disease onset at the age of 2 years (Table 1). They had intellectual and learning disabilities. Both patients exhibited abnormal gaits and had a tendency to fall and stumble due to poor balance. The muscles were stiff, and loss of gross motor functions was also observed, which included difficulty in balancing, inability to sit properly without support, trembling while walking and difficulty in making rapid movements.

In Family RDFA06, the proband IV:3 was a seven-year-old male. He was diagnosed with complicated spastic paraplegia (Table 1). The disease onset was in infancy and was noticeable as early as the age of one year. He had psychomotor developmental delays, severe intellectual disability and stiff muscles. His speech development was poor. He manifested facial hypertonia and had stereotypic laughter. He had contractures of the hands and feet. Hypertonia of the arm and leg muscles was also evident. He showed spasticity of the lower limb muscles. He could not walk unaided. No microcephaly or seizures were observed. There were no reports of problems with vision or hearing loss. A younger affected male sibling (IV:4), described as having a similar phenotype, died at three years of age. Individual IV:1, the affected younger male cousin developed spastic paraplegia symptoms during early infancy and had similar phenotypes to those seen in individual IV:3. However, he presented no dysmorphic facial features.

### Analyses identify and delineate three loss-of-function variants

For family RDHR07, the analyses of variant call file (VCF) exome sequencing data using Franklin did not reveal a likely pathogenic variant in the exons or splice sites among the 27 very rare homozygous variants or a pair of suspected compound heterozygous variants (Supplementary Table 2). Copy number variant analysis of the exome sequence files via ExomeDepth revealed that the patient data lacked the coverage of exons 19 and 20 of NM_025137.4, *SPG11* (Supplementary Fig. 1). Sequencing after amplification across the deletion revealed that multiple regions were deleted from the mutant allele since BLAT analyses aligned the obtained sequence to exon 18, part of intron 18, part of intron 20 and the exon 21 region of NM_025137.4 *SPG11* (Fig. 1D, Supplementary Fig. 2). Thus, exons 19 and 20 were completely deleted, and a portion of intron 18, the entirety of intron 19 and a part of intron 20 were also deleted (Supplementary Fig. 3) corresponding to NC000015.9:g.44894055_44902826del (Table 2).

**Table 2:** Homozygous pathogenic alleles identified in the participants of the three families.

Family ID	*Position	Gene	Change	gnomADv2 AF	SIFT	REVEL	ACMG
RDHR07	15:44894055-44902826	*SPG11*	NC000015.9:g.44894055_449028del	N/A^#^	N/A	N/A	P
ANMD03	8:38103396	*DDHD2*	NM_015214.3, c.985C > T, p.Arg329Ter	0.00001592^#^	N/A	N/A	P
RDFA06	1:114442672	*AP4B1*	NM_001253852.3, c.965-967delACTinsC, p.Tyr322SerfsTer14	0^#^	N/A	N/A	P

*Chromosomal positions according to GRCh37/hg19 assembly. gnomAD, Genome Aggregation Database, AF, Allele frequency, SIFT, Sorting intolerant from Tolerant, REVEL, Rare Exome Variant Ensemble Learner, ACMG, American College of Medical Genetics and Genomics (according to the PP1, PVS1, PM2 criteria), N/A not available or not applicable, P, Pathogenic, ^#^The variants are also absent or extremely rare in the updated gnomADv4 data.

PCR analyses performed in duplicate with the deletion-specific and wild-type-specific set of primers revealed that individuals III:10, III:11 and IV:2 were carriers of the deletion variant; patients IV:9, IV:10 and V:5 were homozygous for the deletion; and individuals IV:4, IV:5, IV:6, IV:11 and V:7 had only the wild-type allele (Fig. 1A, Supplementary Fig. 4). Patients IV:4 and V:7, though displaying some symptoms including spasticity or dystonia similar to their affected cousins (Table 1) did not have the *SPG11* deletion allele, thus demonstrating intrafamilial genetic heterogeneity and indicating a separate cause of their phenotype.

For family ANMD03, exome sequencing data revealed 18 homozygous variants after applying the filtration criteria (Supplementary Table 4). Variants in most of these genes were either predicted benign or were not correlated to the patients’ conditions (Supplementary Table 4). However, the nonsense variant in *DDHD2* (NM_015214.3) c.985C > T;p.(Arg329Ter) was prioritized (Table 2) because it was already known to cause spastic paraplegia (ClinVar VCV000452548.6)^19^. This *DDHD2* variant segregated with the disease phenotype (Fig. 1E).

Finally, for family RDFA06, variant prioritization left 30 rare homozygous or hemizygous gene variants, including frameshift, missense and synonymous changes (Supplementary Table 5). The number of prioritized variants was further reduced to one by discarding those that affected amino acids not conserved in the vertebrates or for which the changes were predicted to be benign by multiple software programs (Supplementary Table 5). A frameshift insertion‒deletion in *AP4B1* (NM_001253852.3), c.965-967delACTinsC;p.(Tyr322SerfsTer14), was the most likely variant (Table 2) that could account for the phenotype and segregated with the disease (Fig. 1F). The parents and the unaffected siblings were heterozygous, and the patients were homozygous for the variant.

All three variants were designated as pathogenic according to the ACMG guidelines (PP1, PVS1, PM2 criteria). These variants were either absent from the public databases or extremely rare (0.00001592 allele frequency). The variants have been deposited in LOVD (https://www.lovd.nl/) with accession numbers 0000945932, 0000945931 and 0000945930, corresponding to *SPG11*, *DDHD2* and *AP4B1,* respectively.

### Exome data analyses identifies multiple large regions of homozygosity

Analyses of the VCF data identified regions of homozygosities (ROH) for all patients which were located on different chromosomes (Supplementary Fig. 5). Specifically, for family RDHR07, patient IV:10, we identified 80 different ROH which were spread throughout the genome except on chromosome 18 (Supplementary Fig. 5). Among these was a ROH on chromosome 15, at coordinates 38.7–57.8 Mb, corresponding to approximately 19 Mb (Supplementary Fig. 6). *SPG11* is located on chromosome 15:44,854,894–44,955,876 and thus the gene coordinates are included in the detected ROH. The parents of the patient IV:10 were unaware of the exact consanguinity relationship. However, the detection of multiple large ROH suggests that the two parents were closely related. For family ANMD03 and family RDFA06, analyses shortlisted 32 and 65 ROH respectively, spread throughout the genome in the two patients who were born to first cousin parents. The ROH were located on chromosomes 1–6, 8–17 and 20–21 for family ANMD03 and all chromosomes except 13 for family RDFA06 (Supplementary Fig. 5). The *DDHD2* and *AP4B1* variants coordinates were included in the detected ROH of ~ 18 Mb on chromosome 8 and ~ 14 Mb on chromosome 1 (Supplementary Fig. 6), corresponding to the data from the respective patients. Our calculations indicated that approximately 7.5%, 5.7% and 9.8% of all exome variants for family RDHR07, ANMD03 and RDFA06, respectively, were located within the regions of homozygosity.

## Discussion

Pakistani population practices consanguineous marriages with a high rate of 60%. Consequently rare recessive disorders are detected^20^ with many patients affected due to inheritance of two copies of the same variant, traceable to the presence of a common ancestor. Variants of many genes have been associated with autosomal recessive HSPs in Pakistani population, which include *ATL1, ALS2, ABHD16A, TFG, AMFR, KY, TECPR2, CYP2U1* and *ZFYVE26*^21–28^*.* In spite of this progress, the incidence of hereditary spastic paraplegia and the genetic etiology remains unknown. We recruited three consanguineous families in which multiple patients were affected by HSPs and completed genetic evaluations. Exome sequencing identified many homozygous variants for each patient which survived the preliminary filtration. However, our data allowed us to clearly associate only one variant for the conditions in the respective families. Still, a modifying role of some of the accompanying predicted benign variants or those with unknown significance cannot be ruled out.

We also found that patients in familyRDHR07 exhibited intrafamilial genetic heterogeneity since the detected variant could not explain the phenotype for two of the five patients. In the remaining three patients for the same family, we discovered an 8.771 kb homozygous deletion within *SPG11*. There are a total of 24 large genomic deletion variants in *SPG11* associated with autosomal recessive spastic paraplegia (http://www.hgmd.cf.ac.uk/ac/gene.php, accessed February 2024). It has been hypothesized that this very large number of genomic rearrangement variants of *SPG11* is due to the presence of repetitive elements as well as recombination hotspots in this chromosomal region^29^.

Affected individuals of the family RDHR07 exhibited very mild intellectual impairment, lower limb spasticity, and weakening of the muscles of the hand. The Babinski sign was also observed in two of the affected patients. A previous study identified an intragenic deletion of 2.6 kb in *SPG11* as a cause of complex spastic paraplegia without a thin corpus callosum^30^, though the latter finding is present in many individuals with *SPG11* variants. A frameshift variant of *SPG11* has also been associated with spastic paraplegia in a Pakistani family^31^. Our study reports the first instance of a large genomic deletion of *SPG11* in patients from Pakistan. Additional research will determine if *SPG11* variants are as commonly present in Pakistani patients with spastic paraplegia as observed in different global populations.

*SPG11* is highly expressed in the nervous system, and its encoded protein spactacsin plays a role in maintaining the cytoskeletal stability and transporting synaptic vesicles^32^. Deletion of *SPG11* exons 19 and 20 from 40 exons gene with multiple intronic regions in the DNA of patients of the current family is expected to disrupt the transcription/splicing^33^ and/or the absence of the two exons could lead to the formation of an aberrant truncated protein. However, what is more likely is that the deletion of the exons from the RNA will introduce a premature stop codon in the open reading frame, perhaps marking the transcript for nonsense mediated decay^34^. In either case, the variant is expected to be a loss of function allele. The knockdown of *spg11* in zebrafish compromises the development of spinal motor axons^35^. A study that evaluated the impact of variants of *SPG11* on forebrain neurons of patients with HSP, revealed the downregulation of axonal-associated genes and decreased neurite complexity^36^.

*DDHD2* encodes a phospholipase which plays a role in membrane trafficking. To date, 21 variants have been described in *DDHD2* (http://www.hgmd.cf.ac.uk/ac/gene.php, accessed February 2024). The variant of *DDHD2*, c.985C > T p.(Arg329Ter), identified in members of the family ANMD03, introduces a stop codon in exon 8 of *DDHD2* (OMIM: 615,003) consisting of a total of 18 exons. This variant results in the formation of a premature termination codon in *DDHD2*. The loss-of-function variant most likely leads to nonsense-mediated decay of the mutant transcript as has been demonstrated for other similar variants of this gene^37^. However, if the protein is synthesized, phospholipase activity will be lost due to the absence of the catalytic domains. The same variant was previously reported in French and Sudanese patients^19,38^ and has additional entries in ClinVar. Individuals affected by *DDHD2* variants have a wide range of phenotypes, including mild to moderate intellectual disability, wide upper and lower limb spasticity, impaired gait, and brisk tendon reflexes^39^. These phenotypes are similar to the symptoms of our patients with the difference that the family ANMD03 patients had severe intellectual disabilities and aggressive behaviors.

An unusual lipid peak was found by cerebral magnetic resonance spectroscopy in patients with *DDHD2* variants, and an accumulation of lipids in the basal ganglia and thalamus was detected^37^. Knockout *Ddhd2*^−/−^ mice exhibit increased triglyceride (TAG) levels in the nervous system. *Ddhd2*^*−/−*^ mice exhibit motor and cognitive deficits, poor rotarod performance and defects in long-term spatial memory^40^. Studies have shown that the central nervous system has a specific pathway for the breakdown of TAGs, and disturbance of this pathway causes an immense accumulation of lipids in neurons and could explain the observation of complicated HSP^40^.

In family RDFA06, a homozygous insertion‒deletion variant of *AP4B1*, c.965-967delACTinsC, p.(Tyr322SerfsTer14), segregated with the disease. This frameshift variant introduces a premature stop codon in the open reading frame. The identified variant is located in exon 5 of a gene which has a total of 10 exons; thus this variant could lead to nonsense-mediated decay (NMD) of the mRNA. If the RNA escapes NMD, important protein domains will be missing in the truncated protein. Another frameshift variant (c.967delT, p.Ser323ArgfsTer18) resulting from deletion of one nucleotide in this codon is known to cause spastic paraplegia^41^.

AP4B1 is one of the four subunits of adaptor protein complex-4 (AP-4). The AP-4 complex is known to play a role in the polarized sorting of cargo in epithelial cells and neurons. The AP-4 complex plays an important role in the transport of α-amino-3-hydroxy-5-methyl-4-isoxazolepropionic acid (AMPA) receptor molecules in the somatodendritic domain of neurons. Mice deficient in the AP-4 complex do not exhibit significant neurological conditions or growth retardation but exhibit poor rotorod performance^42^. In *Ap4b1-*deficient mice, the AMPA glutamate receptor mislocalizes to axons instead of being targeted to the somatodentritic area of neurons^42,43^ and the same phenomenon may perhaps occur in patients with loss of function *AP4B1* variants.

*AP4B1* is ubiquitously expressed in all embryonic and postnatal tissues in humans. The homozygous truncating variants c.487_488insTAT: p.(Glu163ValfsTer2) and c.664delC (p.(Leu222CysfsTer31) of *AP4B1* were first identified to be linked with hereditary spastic paraplegia. The patients were unable to walk or had a waddling gait. All patients exhibited infantile muscular hypotonia, which subsequently involved hypertonia, severe cognitive deficits, speech delays, microcephaly, dysmorphic facial features, short statures and pes planus feet^43,44^. The *AP4B1* frameshift variant detected in the affected individuals of the family RDFA06 resulted in phenotypes similar to those commonly observed in other patients with *AP4B1* variants (OMIM: 614,066) except that hypotonia was not observed.

Nearly 45 variants of *AP4B1* have been identified (HGMD accessed February 2024), including missense, nonsense and frameshift variants. Abnormal or absent AP4B1 protein is predicted to disrupt AP-4 complex formation^45^. Quantitative real-time PCR analysis of patient skin fibroblasts harboring the *AP4B1* c.487_488insTAT p.(Glu163insValTer) variant revealed that the transcript level of *AP4B1* was significantly lower than that detected in the control skin fibroblasts. This result suggested that the variant might cause nonsense mediated decay of mRNA.

## Conclusions

In conclusion, our study identified the variants of three different genes associated with HSP. This research broadens the genetic spectrum of *SPG11* and *AP4B1.* Additional studies will pinpoint founder mutations and frequently mutated genes in spastic paraplegia in Pakistan.

### Supplementary Information

Supplementary Information.

## Data Availability

Variants have been deposited in LOVD (https://www.lovd.nl/) with accession numbers 0000945932, 0000945931 and 0000945930, corresponding to *SPG11*, *DDHD2* and *AP4B1,* respectively. All the data have been presented in the article or as supplementary material. Additional supporting material is available from the corresponding author upon reasonable request.
