# Supplementary material for: Identification and analyses of exonic and copy number variants in spastic paraplegia
Source: Sci Rep. 2024 Jun 21;14:14331. doi: 10.1038/s41598-024-64922-8 (PMC11192879; doi:10.1038/s41598-024-64922-8)
Supplement: Supplementary file 1 — Supplementary Information. [file 41598_2024_64922_MOESM1_ESM.docx]

**Supplementary Tables and Figures**

**Identification and analyses of exonic and copy number variants in spastic paraplegia**

Anum Shafique^1*^, Ayesha Nadeem^1*^, Faiza Aslam^1*^, Humera Manzoor^1^, Muhammad Noman^1^,

Elizabeth Wohler^2^, P Dane Witmer^2^, Nara Sobreira^2^, Sadaf Naz^1**^

^1^School of Biological Sciences, University of the Punjab, Quaid-e-Azam Campus, Lahore 54590, Pakistan

^2^McKusick-Nathans Department of Genetic Medicine, Baylor Hopkins Center for Mendelian Genomics Baltimore, MD, USA.

*AS, AN and FA contributed equally to this manuscript

****Correspondence**

Sadaf Naz, PhD, School of Biological Sciences, University of the Punjab, Quaid-e-Azam Campus, Lahore 54590, Pakistan Phone: +92-42-99231819, Email: naz.sbs@pu.edu.pk

**Supplementary Table 1**: Sequences of the primers used in this study

| **Name** | **Sequences** | **Product Size (bp)** |
| --- | --- | --- |
| SPG11wt-F | AGAGTATAGCCTTTGCGGACA | 483 |
| SPG11wt-R | TTCAATTCTGTCTTTGCGAACT |  |
| SPG11del-F | TCCCACCAATCAGGCCAGTGTAAGC | 1600 |
| SPG11del-R | TCCTGGACCAGAAAAGTACCAAATGCAAA |  |
| DDHD2-F | TTGATTACCACTTACCTCTCTGTCT | 591 |
| DDHD2-R | GACCACTTCTAGACCTCTGAATTTC |  |
| AP4B1-F | TGGCTGGTCTAGGTTCTCAAG | 400 |
| AP4B1-R | GGGAGCTACCAAACTTTTTCTG |  |

**Supplementary Table 2:** Rare homozygous variants observed for family RDHR07 in the VCF data

| ***Position** | **Gene** | **Change** | **gnomAD AF** | **SIFT** | **REVEL** | **Comments** |
| --- | --- | --- | --- | --- | --- | --- |
| 1:1354589 | *ANKRD65* | NM_001145210.2, c.1091G>A, p.Arg364Gln | 6.37E-05 | 0.052, U | 0.032, B | All software, except one, predicted the variant to be benign. The amino acid has not been conserved through evolution |
| 1:6309542 | *GPR153* | NM_207370.4, c.1686C>T, p.Arg562Arg | 0 | N/A | N/A | Synonymous change, Not predicted to affect splicing |
| 1:8420199 | *RERE* | NM_001042681.2, c.3368C>A, p.Thr1123Asn | 0 | 0.002, U | 0.254, B | All software, except one, predicted the variant to be benign. The amino acid has not been conserved through evolution |
| 1:16053861 | *PLEKHM2* | NM_015164.4, c.1294C>A, p.Pro432Thr | 6.39E-05 | 0.046, U | 0.034, B | All software, except one, predicted the variant to be benign. The amino acid has not been conserved through evolution |
| 1:28199088 | *THEMIS2* | NM_001105556.3, c.6G>T, p.Glu2Asp | 0 | 0, D | 0.06, B | All software, except one, predicted the variant to be benign. Asp is the wild type amino acid in some mammalian species |
| 1:67672567 | *IL23R* | NM_144701.2, c.653-14_653-5dupTTTTTTTTTT | 0 | N/A | N/A | Intronic variant , Not predicted to affect splicing |
| 1:154091294 | *NUP210L* | NM_207308.2, c.1327-10G>C | 9.63E-05 | N/A | N/A | Intronic variant at a non-conserved position, Not predicted to affect splicing |
| 1:16385163 | *FAM131C* | NM_182623.3, c.612G>A, p.Gln204Gln | 0.001664 | N/A | N/A | Synonymous change, Not predicted to affect splicing |
| 1:196759209 | *CFHR3* | NM_021023.5, c.648T>C, p.Ile216Ile | 0 | N/A | N/A | Synonymous change, Not predicted to affect splicing |
| 2:232220601 | *ARMC9* | NM_001352754.1, c.2086A>G, p.Arg696Gly | 0.000223 | N/A | 0.025, B | Classified as benign in ClinVar VCV000862706.8 for OMIM 617622 |
| 1:24434998 | *MYOM3* | NM_152372.4, c.129C>G, p.Gly43Gly | 0 | N/A | N/A | Synonymous change, Not predicted to affect splicing |
| 1:26299080 | *PAFAH2* | NM_000437.4, c.1053G>T, p.Arg351Arg | 0 | N/A | N/A | Synonymous change, Not predicted to affect splicing |
| 3:69236999 | *FRMD4B* | NM_015123.3, c.1841C>A, p.Pro614His | 3.20E-05 | 0.259, D | 0.49,U | All software, except one, predicted the variant to be benign. The amino acid has not been conserved through evolution |
| 4:170652863 | *HPF1* | NM_017867.3, c.901G>A, p.Gly301Ser | 9.56E-05 | 0, D | 0.575, U | Also homozygous in two unaffected ethnically matched individuals |
| 5:56777878 | *ACTBL2* | NM_001017992.4, c.657C>T, p.Tyr219Tyr | 0.000701 | N/A | N/A | Synonymous change, Not predicted to affect splicing |
| 7:121653048 | *PTPRZ1* | NM_002851.3, c.3948T>C, p.Val1316Val | 0 | N/A | N/A | Synonymous change, Not predicted to affect splicing |
| 8:38065274 | *BAG4* | NM_004874.4, c.623C>T, p.Pro208Leu | 3.19E-05 | 0.001, U | 0.299, U | All software, except one, predicted the variant to be benign or of unknown significance. The amino acid has not been conserved through evolution |
| 8:94738624 | *FAM92A* | NM_145269.4, c.660T>G, p.Val220Val | 0 | N/A | N/A | Synonymous change, Not predicted to affect splicing |
| 10:26802583 | *APBB1IP* | NM_019043.4, c.807C>T, p.Asn269Asn | 6.38E-05 | N/A | N/A | Synonymous change, Not predicted to affect splicing |
| 12:30863339 | *CAPRIN2* | NM_001002259.2, c.2881G>A, p.Asp961Asn | 0 | 0.001, U | 0.323, U | All software, except one, predicted the variant to be benign or of uncertain significance. |
| 12:52307540 | *ACVRL1* | NM_000020.2, c.511G>A, p.Asp171Asn | 3.19E-05 | 0.084, B | 0.439, U | Although predicted deleterious by 2/6 software, the affected amino acid has not been conserved through evolution. |
| 12:52826941 | *KRT75* | NM_004693.3, c.594C>A, p.Pro198Pro | 0 | N/A | N/A | Synonymous change, Not predicted to affect splicing |
| 12:57036248 | *ATP5F1B* | NM_001686.4, c.1068T>C, p.Ser356Ser | 0 | N/A | N/A | Synonymous change, Not predicted to affect splicing |
| 13:51530566 | *RNASEH2B* | NM_024570.3, c.895A>G, p.Thr299Ala | 0 | 0.218, B | 0.184, B | Classified as likely benign in ClinVar, VCV000705177.11 for OMIM 610181 |
| 15:54307647 | *UNC13C* | NM_001080534.2, c.2547C>T, p.Asp849Asp | 0 | N/A | N/A | Synonymous change, Not predicted to affect splicing |
| 15:91475015 | *HDDC3* | NM_001286451.1, c.328C>G, p.His110Asp | 0 | 0.254, B | 0.095, B | All software, except one, predicted the variant to be benign. The amino acid has not been conserved through evolution |
| 16:28145189 | *XPO6* | NM_015171.3, c.1509C>T, p.Leu503Leu | 9.56E-05 | N/A | N/A | Synonymous change, Not predicted to affect splicing |

*Chromosomal position according to GRCh37/hg19 assembly, gnomAD, Genome Aggregation Database, AF, Allele frequency, SIFT, SIFT, Sorting intolerant from Tolerant (score and prediction), REVEL, Rare Exome Variant Ensemble Learner (score and prediction), N/A not available or not applicable, D, deleterious or damaging, B, benign, U, unknown. Predictions were considered from SIFT, REVEL (shown) as well as PolyPhen2, FATHMM, DANN and Splice AI (not shown). For synonymous variants, splicing scores from dbscSNV Ada and dbscSNV RF were also considered. ClinVar, Clinical Variant, OMIM, Online Mendelian Inheritance in Man.

**Supplementary Table 3:** Putative compound heterozygous variants shortlisted for family RDHR07in the VCF data

| ***Position** | **Gene** | **Change** | **gnomAD AF** | **SIFT** | **REVEL** | **Comments** |
| --- | --- | --- | --- | --- | --- | --- |
| 8:143399919 | *TSNARE1* | NM_145003.5,  c.970C>T,  p.Arg324Cys | 0.000159 | 0.002, U | 0.082, B | All software, except one, predicted the variant to be benign or of unknown significance. The amino acid has not been conserved through evolution |
| 8:143381954 | *TSNARE1* | NM_145003.5,  c.1183G>A,  p.Gly395Arg | 0 | 0.016, U | 0.129, B | All software, except one, predicted the variant to be benign or of unknown significance. The amino acid has not been conserved through evolution |

*Chromosomal position according to GRCh37/hg19 assembly, gnomAD, Genome Aggregation Database, AF, Allele frequency, SIFT, SIFT, Sorting intolerant from Tolerant (score and prediction), REVEL, Rare Exome Variant Ensemble Learner (score and prediction), B, benign, U, unknown. Predictions were considered from SIFT, REVEL (shown) as well as PolyPhen2, FATHMM, DANN and Splice AI (not shown).

**Supplementary Table 4**: Rare homozygous variants observed for family ANMD03

| ***Position** | **Gene** | **Change** | **gnomAD AF** | **SIFT** | **REVEL** | **Comments** |
| --- | --- | --- | --- | --- | --- | --- |
| 1:82456820 | *ADGRL2* | NM_001366005.2, c.4401A>C, p.Thr1467Thr | 0 | N/A | N/A | Synonymous change, Not predicted to affect splicing |
| 1:91403496 | *ZNF644* | NM_201269.3, c.3234T>G, p.Val1078Val | 0 | N/A | N/A | Synonymous change, Not predicted to affect splicing |
| 1:95657143 | *TLCD4* | NM_152487.3, c.511A>G, p.Lys171Glu | 0 | 0.282,B | 0.458, U | All software, except one, predicted the variant to be benign or of unknown significance. The amino acid has not been conserved through evolution |
| 1:100361929 | *AGL* | NM_000642.3, c.3347G>A, p.Arg1116His | 3.18E-05 | 0.001,U | 0.765, D | Although predicted predominantly deleterious, the amino acid has not been conserved in evolution |
| 4:162577600 | *FSTL5* | NM_020116.5, c.774T>G, p.Thr258Thr | 0 | N/A | N/A | Synonymous change, Not predicted to affect splicing |
| 5:140209281 | *PCDHA6* | NM_031849.3, c.1602+3G>T | 0 | N/A | N/A | Intronic variant at a non-conserved position, Not predicted to affect splicing |
| 5:140558400 | *PCDHB8* | NM_019120.5, c.785T>C, p.Val262Ala | 0 | 0.023,U | 0.328, U | All software, except one, predicted the variant to be benign or of unknown significance. The amino acid has not been conserved through evolution |
| 5:140625305 | *PCDHB15* | NM_018935.4, c.159G>T, p.Gly53Gly | 0 | N/A | N/A | Synonymous change, Not predicted to affect splicing |
| 6:86199291 | *NT5E* | NM_002526.4, c.1184G>A, p.Arg395Gln | 0 | 0.038,U | 0.582, U | Predicted deleterious by only 2/6 software. Variants of this gene cause Calcification of joints and arteries (OMIM 211800) |
| 8:22006031 | *LGI3* | NM_139278.4, c.1289G>A, p.Arg430His | 3.19E-05 | 0.113,B | 0.307, U | All software, except one, predicted the variant to be benign. The amino acid has not been conserved through evolution |
| **8:38103396** | ***DDHD2*** | **NM_015214.3, c.985C>T, p.Arg329Ter** | 0.00001592 | **N/A** | **N/A** | **Known pathogenic variant, ClinVar VCV000452548.8. Segregated with the phenotype** |
| 9:100862340 | *TRIM14* | NM_014788.4, c.410C>T, p.Thr137Met | 0 | 0.152,B | 0.11, B | All software predicted the variant to be benign. The amino acid Met is also present in many mammals at the corresponding position |
| 9:114355266 | *PTGR1* | NM_001146108.2, c.153-15_153-5delTTTTTTTTTTT | 0 | N/A | N/A | Intronic variant, Not predicted to affect splicing. Also homozygous in two unrelated ethnically matched individuals without the corresponding phenotype |
| 12:109185905 | *SSH1* | NM_001161331.1, c.2083C>T, p.Gln695Ter | 9.56E-05 | N/A | N/A | Variants of this gene cause actinic porokeratosis PMID15459975. This variant is predicted to truncate the last seven amino acids of the protein |
| 12:112513588 | *NAA25* | NM_024953.4, c.670T>C, p.Leu224Leu | 0 | N/A | N/A | Synonymous change, Not predicted to affect splicing |
| 14:92361409 | *FBLN5* | NM_006329.4, c.387C>T, p.Asp129Asp | 6.37E-05 | N/A | N/A | Synonymous change, Not predicted to affect splicing |
| 20:18143041 | *KAT14* | NM_020536.5, c.1123G>A  p.Asp375Asn | 0 | 0.305, B | 0.117, B | All software, except one, predicted the variant to be benign or of unknown significance. |
| 21:44171229 | *PDE9A* | NM_002606.3, c.657C>T, p.Thr219Thr | 0 | N/A | N/A | Synonymous change, Not predicted to affect splicing |

*Chromosomal position according to GRCh37/hg19 assembly. gnomAD, Genome Aggregation Database, AF, Allele frequency, SIFT, Sorting intolerant from Tolerant (score and prediction), REVEL, Rare Exome Variant Ensemble Learner (score and prediction), N/A not available or not applicable, D, deleterious or damaging, B, benign, U, unknown. Predictions were considered from SIFT, REVEL (shown) as well as PolyPhen 2, FATHMM, DANN and Splice AI (not shown). For synonymous and other splicing variants, scores from dbscSNV Ada and dbscSNV RF were also considered. ClinVar, Clinical Variant, PMID, PubMed Identifier, OMIM, Online Mendelian Inheritance in Man. Bold lettering indicates the information for the selected variant which was checked for segregation.

**Supplementary Table 5:** Rare homozygous and hemizygous variants observed for family RDFA06

| ***Position** | **Gene** | **Change** | **gnomAD AF** | **SIFT** | **REVEL** | **Comments** |
| --- | --- | --- | --- | --- | --- | --- |
| **1:114442672** | ***AP4B1*** | **NM_001253852.3,**  **c.965-967delACTinsC,**  **p.Tyr322SerfsTer14** | **0** | **N/A** | **N/A** | **ACMG, pathogenic variant in a known gene for spastic paraplegia, OMIM 614066, Segregated with the phenotype** |
| 1:183190055 | *LAMC2* | NM_005562.2,  c.599C>A,  p.Ala200Glu | 0.000287 | 1, B | 0.103, B | All software predicted the variant to be benign. The amino acid has not been conserved through evolution. ClinVar SCV001062136, benign for OMIM 619785 |
| 1:186037119 | *HMCN1* | NM_031935.3,  c.7859G>A,  p.Arg2620Gln | 0 | 0.337, B | 0.142, B | All software, except one, predicted the variant to be benign or of unknown significance. The amino acid has not been conserved through evolution |
| 3:127339654 | *MCM2* | NM_004526.4,  c.2379G>A,  p.Met793Ile | 0 | 0.33, B | 0.258, B | All software, except one, predicted the variant to be benign or of unknown significance. |
| 4:151773935 | *LRBA* | NM_001364905.1,  c.2927C>T,  p.Thr976Met | 3.18E-05 | 0.258, B | 0.055, B | All software predicted the variant to be benign or of unknown significance. The amino acid has not been conserved through evolution. ClinVar VCV000658696.7, Variant of unknown significance for OMIM 614700 |
| 7:148712075 | *PDIA4* | NM_004911.5,  c.535A>G,  p.Thr179Ala | 0 | 0.103, B | 0.136, B | All software, except one, predicted the variant to be benign or of unknown significance. The amino acid has not been conserved through evolution |
| 7:154767531 | *PAXIP1* | NM_007349.3,  c.949C>G,  p.Gln317Glu | 0 | 0.106, B | 0.115, B | All software, except one, predicted the variant to be benign or of unknown significance. The amino acid has not been conserved through evolution |
| 8:22481871 | *BIN3* | NM_018688.5,  c.344G>C,  p.Gly115Ala | 0 | 0.319, B | 0.086, B | All software predicted the variant to be benign or of unknown significance. The amino acid has not been conserved through evolution |
| 8:25346210 | *CDCA2* | NM_152562.4,  c.1671+5G>T | 0 | N/A | N/A | This variant was predicted to affect splicing by 1/3 software and to be benign by the other two. The nucleotide affected by the variant has not been conserved through evolution |
| 8:28214250 | *ZNF395* | NM_018660.3,  c.520C>T,  p.Leu174Leu | 0 | N/A | N/A | Synonymous variant, None of the software used predicted any effect on splicing |
| 8:35588536 | *UNC5D* | NM_080872.4,  c.1688G>C,  p.Ser563Thr | 3.19E-05 | 0.001, U | 0.499, U | Predicted deleterious by 2/6 software but a conservative change and also not predicted to affect splicing |
| 8:42587170 | *CHRNB3* | NM_000749.4,  c.720C>T,  p.Leu240Leu | 3.18E-05 | N/A | N/A | Synonymous variant, None of the three software used predicted any effect on splicing |
| 9:14851317 | *FREM1* | NM_144966.5,  c.1117C>T,  p.Pro373Ser | 0 | 0.12, B | 0.414, U | All but two software predicted the variant to be benign or of unknown significance. The amino acid has not been conserved through evolution |
| 9:26925818 | *PLAA* | NM_001031689.3,  c.869+5T>G | 0 | N/A | N/A | This variant was predicted benign and to have no effect on splicing by all software used. The nucleotide affected by the variant has not been conserved through evolution |
| 12:102591522 | *PMCH* | NM_002674.4,  c.27T>C,  p.Tyr9Tyr | 0 | N/A | N/A | Synonymous variant, None of the software used predicted any effect on splicing |
| 15:41276099 | *INO80* | NM_017553.3,  c.4098G>A,  p.Leu1366Leu | 0 | N/A | N/A | Synonymous variant, None of the software used predicted any effect on splicing |
| 15:48905297 | *FBN1* | NM_000138.4,  c.165-8T>C | 0 | N/A | N/A | This variant was predicted benign and to have no effect on splicing by all software used. The nucleotide affected by the variant has not been conserved through evolution |
| 16:2230811 | *CASKIN1* | NM_020764.3,  c.2558C>G,  p.Pro853Arg | 0.000829 | 0.055, U | 0.085, B | All but one software predicted the variant to be benign or of unknown significance. The amino acid has not been conserved through evolution |
| 17:48918331 | *WFIKKN2* | NM_175575.5,  c.1682T>C,  p.Met561Thr | 0 | 0.012, U | 0.077, B | All but one software predicted the variant to be benign or of unknown significance. The amino acid has not been conserved through evolution |
| 19:55349051 | *KIR2DS4* | NM_012314.6,  c.91T>C,  p.Phe31Leu | 0 | N/A | N/A | Only PolyPhen2 and Splice AI predictions were available, which were benign. The amino acid has not been conserved through evolution |
| 19:55349061 | *KIR2DS4* | NM_012314.6,  c.101T>A,  p.Leu34His | 0 | N/A | N/A | Only PolyPhen2 and Splice AI predictions were available, which were benign. The amino acid has not been conserved through evolution |
| 22:28501481 | *TTC28* | NM_001145418.1,  c.3093C>T,  p.Asn1031Asn | 0.001466 | N/A | N/A | Synonymous variant, None of the software used predicted any effect on splicing |
| 22:30951503 | *GAL3ST1* | NM_001318104.2,  c.709G>A,  p.Glu237Lys | 0.000255 | 0.348, B | 0.109, B | All but one software predicted the variant to be benign or of unknown significance. The amino acid has not been conserved through evolution |
| 22:31010356 | *TCN2* | NM_000355.3,  c.448C>G,  p.Pro150Ala | 0 | 0, D | 0.279, B | All but two software predicted the variant to be benign or of unknown significance. The amino acid has not been conserved through evolution |
| 22:31536233 | *PLA2G3* | NM_015715.5,  c.108C>T,  p.Ala36Ala | 0 | N/A | N/A | Synonymous variant, None of the software used predicted any effect on splicing |
| 22:35719548 | *TOM1* | NM_005488.3,  c.426G>T,  p.Glu142Asp | 0 | 0.11, B | 0.193, B | Predicted deleterious by 2/6 software but a conservative change and also not predicted to affect splicing |
| 22:37333941 | *CSF2RB* | NM_000395.3,  c.2091G>A,  p.Met697Ile | 0.000128 | 0.562, B | 0.13, B | All software predicted the variant to be benign or of unknown significance. The amino acid has not been conserved through evolution |
| 22:37578239 | *C1QTNF6* | NM_031910.4,  c.826G>A,  p.Glu276Lys | 0 | 0.076, U | 0.056, B | All but one software predicted the variant to be benign or of unknown significance. The amino acid has not been conserved through evolution |
| 22:42609096 | *TCF20* | NM_005650.3,  c.2216A>G,  p.His739Arg | N/A | 0.002, U | 0.192, B | All but one software predicted the variant to be benign or of unknown significance. The amino acid has not been conserved through evolution |
| X:153278669 | *IRAK1* | NM_001569.4,  c.1755C>T,  p.Pro585Pro | 0 | N/A | N/A | Synonymous variant, None of the software used predicted any effect on splicing |

*Chromosomal position according to GRCh37/hg19 assembly, gnomAD, Genome Aggregation Database, AF, Allele frequency, SIFT, Sorting intolerant from Tolerant (score and prediction), REVEL, Rare Exome Variant Ensemble Learner (score and prediction), ACMG, American College of Medical Genetics and Genomics, N/A not available or not applicable, D, deleterious or damaging, B, benign, U, unknown. Predictions were considered from SIFT, REVEL (shown) as well as PolyPhen2, FATHMM, DANN and Splice AI (not shown). For synonymous and other splicing variants, scores from dbscSNV Ada and dbscSNV RF were also considered. ClinVar, Clinical variation. OMIM, Online Mendelian Inheritance for Man. Bold lettering indicates the information for the selected variant which was checked for segregation.


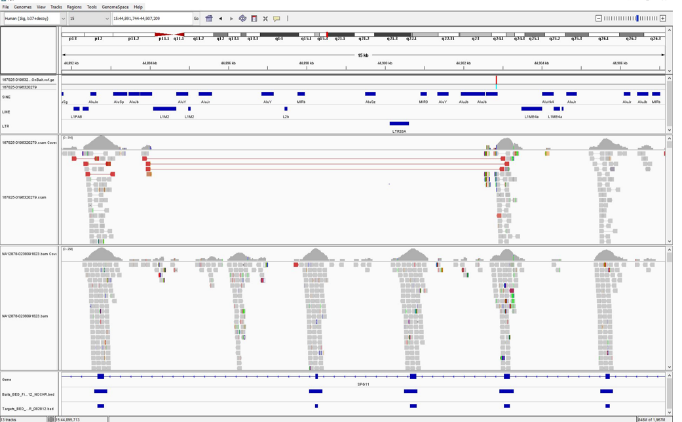


**Supplementary Fig. 1.** BAM files visualization in IGV server. NM_025137.4, SPG11 Exons 19 and 20 showed complete absence of reads in the top sample (shown with long red horizontal lines) from the patient of family RDHR07 as compared to the presence of reads from unrelated individual (bottom row).

## Alignment of deletion allele to chr15:44892695-44903134 (hg19)

**Aligned section 1 (deletion allele to genomic)**

*SPG11* deletion allele sequence, top strand, starting from position 1 (ends at 310)

*SPG11* genomic sequence chr15, hg19, lower strand, start position 44903134

00000001 tcccaccaatcaggccagtgtaagcagtatgctattggaaggacataccc 00000050

<<<<<<<< |||||||||||||||||||||||||||||||||||||||||||||||||| <<<<<<<<

44903134 tcccaccaatcaggccagtgtaagcagtatgctattggaaggacataccc 44903085

00000051 tcctggcccttgctactacaatgtattctcctgggggtgtcagtcaggta 00000100

<<<<<<<< |||||||||||||||||||||||||||||||||||||||||||||||||| <<<<<<<<

44903084 tcctggcccttgctactacaatgtattctcctgggggtgtcagtcaggta 44903035

00000101 tggatagcactttatgacaaaataggactgatttttaaatttagcagaat 00000150

<<<<<<<< |||||||||||||||||||||||||||||||||||||||||||||||||| <<<<<<<<

44903034 tggatagcactttatgacaaaataggactgatttttaaatttagcagaat 44902985

00000151 tatctatggattgtctagatctcagctgatattaaaatataactatattc 00000200

<<<<<<<< |||||||||||||||||||||||||||||||||||||||||||||||||| <<<<<<<<

44902984 tatctatggattgtctagatctcagctgatattaaaatataactatattc 44902935

00000201 aagtatttcattagattaaagagcagaggataaggctgaatttaaaaatt 00000250

<<<<<<<< |||||||||||||||||||||||||||||||||||||||||||||||||| <<<<<<<<

44902934 aagtatttcattagattaaagagcagaggataaggctgaatttaaaaatt 44902885

00000251 ttatatctcggcccggtgcagtggctcactcctgtaatcgcagcactttg 00000300

<<<<<<<< ||||||||||||||||||||||||||||||||||||||| |||||||||| <<<<<<<<

44902884 ttatatctcggcccggtgcagtggctcactcctgtaatcccagcactttg 44902835

00000301 ggaggccgag 00000310

<<<<<<<< |||||||||| <<<<<<<<

44902834 ggaggccgag 44902825

There is no match of deletion allele sequence to chr15:44894055-44902826 which indicates that the section is deleted from the mutant allele.

**Aligned section 2 (deletion allele to genomic)**

*SPG11* deletion allele sequence, top strand, starting from position 311 (ends at 1621)

*SPG11* genomic sequence chr15, hg19, lower strand, start position 44894005

00000311 gtgggcagatcacccgaggccaggagttcgagaccagcctggccaacatg 00000360

<<<<<<<< |||||||||||||||||||||||||||||||||||||||||||||||||| <<<<<<<<

44894005 gtgggcagatcacccgaggccaggagttcgagaccagcctggccaacatg 44893956

00000361 gcgaaaccccgtctctactgtaagtacaaaaattagctaggtgtggtggc 00000410

<<<<<<<< |||||||||||||||||||||||||||||||||||||||||||||||||| <<<<<<<<

44893955 gcgaaaccccgtctctactgtaagtacaaaaattagctaggtgtggtggc 44893906

00000411 acgtgcctctaagtcctagctacttaggaggctaaggcaggagaatcact 00000460

<<<<<<<< |||||||||||||||||||||||||||||||||||||||||||||||||| <<<<<<<<

44893905 acgtgcctctaagtcctagctacttaggaggctaaggcaggagaatcact 44893856

00000461 tgaacccaggaggcggaagttgcagtgagctgagattgtgccatggcact 00000510

<<<<<<<< |||||||||||||||||||||||||||||||||||||||||||||||||| <<<<<<<<

44893855 tgaacccaggaggcggaagttgcagtgagctgagattgtgccatggcact 44893806

00000511 ccagcctagacagccgagcaagattctatttaaaaaaaaaaaaaaaaaaa 00000560

<<<<<<<< |||||||||||||||||||||||||||||||||||||||||||||||||| <<<<<<<<

44893805 ccagcctagacagccgagcaagattctatttaaaaaaaaaaaaaaaaaaa 44893756

00000561 gaagaggctgggcgcacgcatgtaatcctagcactctgggaggccaaggc 00000610

<<<<<<<< |||||||||||||||||||||||||||||||||||||||||||||||||| <<<<<<<<

44893755 gaagaggctgggcgcacgcatgtaatcctagcactctgggaggccaaggc 44893706

00000611 aggtggattgcttgagctcaggattttgagaccagcctgggcaacatggt 00000660

<<<<<<<< |||||||||||||||||||||||||||||||||||||||||||||||||| <<<<<<<<

44893705 aggtggattgcttgagctcaggattttgagaccagcctgggcaacatggt 44893656

00000661 gaaatctcatctctaccaaaaatactaaagaaagctgggtgtggtggcat 00000710

<<<<<<<< |||||||||||||||||||||||||||||||||||||||||||||||||| <<<<<<<<

44893655 gaaatctcatctctaccaaaaatactaaagaaagctgggtgtggtggcat 44893606

00000711 gcacctgtggtcccagctacttgggaggctgaagtgggaggatcacctga 00000760

<<<<<<<< |||||||||||||||||||||||||||||||||||||||||||||||||| <<<<<<<<

44893605 gcacctgtggtcccagctacttgggaggctgaagtgggaggatcacctga 44893556

00000761 gcccaggaagtggagattgcagtgagccaagattatgccactgcactcca 00000810

<<<<<<<< |||||||||||||||||||||||||||||||||||||||||||||||||| <<<<<<<<

44893555 gcccaggaagtggagattgcagtgagccaagattatgccactgcactcca 44893506

00000811 gtctgggcgacagagccagaccctgtcttaaaaaaaaaaataaaaaatca 00000860

<<<<<<<< |||||||||||||||||||||||||||||||||||||||||||||||||| <<<<<<<<

44893505 gtctgggcgacagagccagaccctgtcttaaaaaaaaaaataaaaaatca 44893456

00000861 attatgttgtatactttaaatatatacaattcttaaataagttgctcaag 00000910

<<<<<<<< |||||||||||||||||||||||||||||||||||||||||||||||||| <<<<<<<<

44893455 attatgttgtatactttaaatatatacaattcttaaataagttgctcaag 44893406

00000911 gtcacgcagctagtggtgtcaagaccaggaatctcacacttttttttgtt 00000960

<<<<<<<< |||||||||||||||||||||||||||||||||||||||||||||||||| <<<<<<<<

44893405 gtcacgcagctagtggtgtcaagaccaggaatctcacacttttttttgtt 44893356

00000961 tttttgaaatgaagtttcactcttgttgcccaggctggagtgcaatggcg 00001010

<<<<<<<< |||||||||||||||||||||||||||||||||||||||||||||||||| <<<<<<<<

44893355 tttttgaaatgaagtttcactcttgttgcccaggctggagtgcaatggcg 44893306

00001011 cgatcttggctcaccacaacctctgcctcccaggttcaagtgattctcct 00001060

<<<<<<<< |||||||||||||||||||||||||||||||||||||||||||||||||| <<<<<<<<

44893305 cgatcttggctcaccacaacctctgcctcccaggttcaagtgattctcct 44893256

00001061 gcctcagtctcccaagtagctgggattacaggcatgcaccgccacatctg 00001110

<<<<<<<< |||||||||||||||||||||||||||||||||||||||||||||||||| <<<<<<<<

44893255 gcctcagtctcccaagtagctgggattacaggcatgcaccgccacatctg 44893206

00001111 gctaattttgtatttttagtagagacggggtttctccaacttggtcaggc 00001160

<<<<<<<< |||||||||||||||||||||||||||||||||||||||||||||||||| <<<<<<<<

44893205 gctaattttgtatttttagtagagacggggtttctccaacttggtcaggc 44893156

00001161 tggtctcaactcttgacctcaggtcatccgcctgccttagtctcccaaag 00001210

<<<<<<<< |||||||||||||||||||||||||||||||||||||||||||||||||| <<<<<<<<

44893155 tggtctcaactcttgacctcaggtcatccgcctgccttagtctcccaaag 44893106

00001211 tgctgggattacaggcgtgagccaccgtacccggctgaagcatcatttct 00001260

<<<<<<<< |||||||||||||||||||||||||||||||||||||||||||||||||| <<<<<<<<

44893105 tgctgggattacaggcgtgagccaccgtacccggctgaagcatcatttct 44893056

00001261 taccagagtttttatgcaacttctcaggtacacatctttgtataaaatga 00001310

<<<<<<<< |||||||||||||||||||||||||||||||||||||||||||||||||| <<<<<<<<

44893055 taccagagtttttatgcaacttctcaggtacacatctttgtataaaatga 44893006

00001311 aggctacctctatacactgaagtaaaataaatcagattttggtagattca 00001360

<<<<<<<< |||||||||||||||||||||||||||||||||||||||||||||||||| <<<<<<<<

44893005 aggctacctctatacactgaagtaaaataaatcagattttggtagattca 44892956

00001361 aagtgatacgtagtgatgcaaaataattacagcaaccagttttatcatta 00001410

<<<<<<<< |||||||||||||||||||||||||||||||||||||||||||||||||| <<<<<<<<

44892955 aagtgatacgtagtgatgcaaaataattacagcaaccagttttatcatta 44892906

00001411 gagagttccatgtgcaaatctgaaattaattatggtgatatacagataat 00001460

<<<<<<<< |||||||||||||||||||||||||||||||||||||||||||||||||| <<<<<<<<

44892905 gagagttccatgtgcaaatctgaaattaattatggtgatatacagataat 44892856

00001461 taatattgttttactttccccctagatgcatggagtcatctcccacattt 00001510

<<<<<<<< |||||||||||||||||||||||||||||||||||||||||||||||||| <<<<<<<<

44892855 taatattgttttactttccccctagatgcatggagtcatctcccacattt 44892806

00001511 ctctagccctgacctggttaataaatatgctatagtggaacgtctgaatt 00001560

<<<<<<<< |||||||||||||||||||||||||||||||||||||||||||||||||| <<<<<<<<

44892805 ctctagccctgacctggttaataaatatgctatagtggaacgtctgaatt 44892756

00001561 ttgcttattatttacataatgggcggccatcatttgcatttggtactttt 00001610

<<<<<<<< |||||||||||||||||||||||||||||||||||||||||||||||||| <<<<<<<<

44892755 ttgcttattatttacataatgggcggccatcatttgcatttggtactttt 44892706

00001611 ctggtccagga 00001621

<<<<<<<< ||||||||||| <<<<<<<<

44892705 ctggtccagga 44892695

**Supplementary Fig. 2**. BLAT result of the sequence determined from the *SPG11* mutant allele (top rows) against the chromosome 15, hg19 genomic sequence (lower rows). chr15:44894055-44902826 did not align and is thus deleted from the patient’s DNA. Please see the Supplementary Fig. 3 for a schematic review of deleted introns and exons.

.


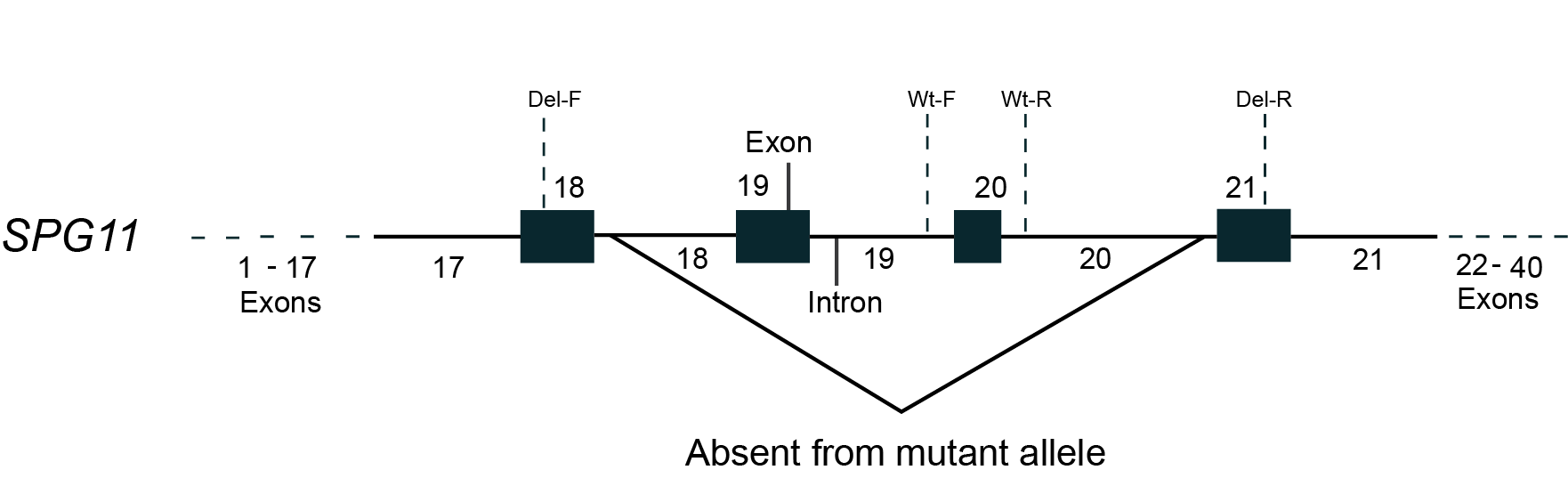


**Supplementary Fig. 3.** Schematic representation of the *SPG11* deletion. Partial *SPG11* gene (with exons 18-21) is shown. Exons are drawn as black boxes, while introns are represented by horizontal lines. Primer locations and product sizes for the wild-type (Wt-F &Wt-R) and the variant *SPG11* (Del-F & Del-R) alleles amplification are indicated with dashed vertical lines. The former primer pairs amplified only the 483 bp wild-type *SPG11* allele, while the latter pair amplified a 1621 bp product only from the mutant allele. This allele was determined by sequencing to lack a portion of intron 18, all of exon 19, intron 19, exon 20 and a part of intron 20. This deleted portion is indicated with two converging slanted lines.


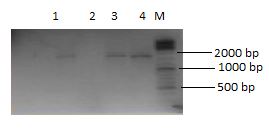


1 2 3 4 5 M

A

B

C

D

E


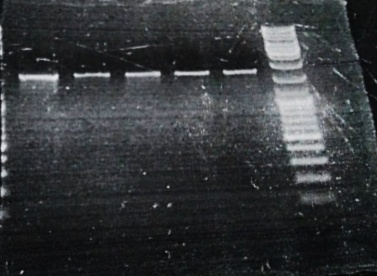


2000 bp

1000 bp

500bp

1 2 3 4 5 6 7 M

**
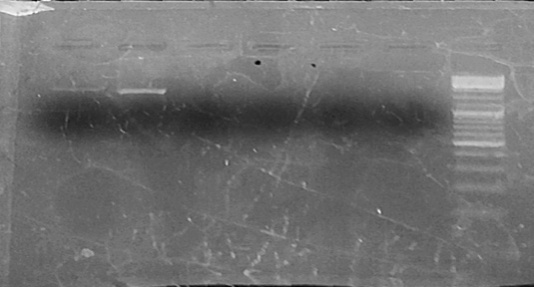
**

2000 bp

1000 bp

500 bp

1 2 3 4 5 6 7 8 M


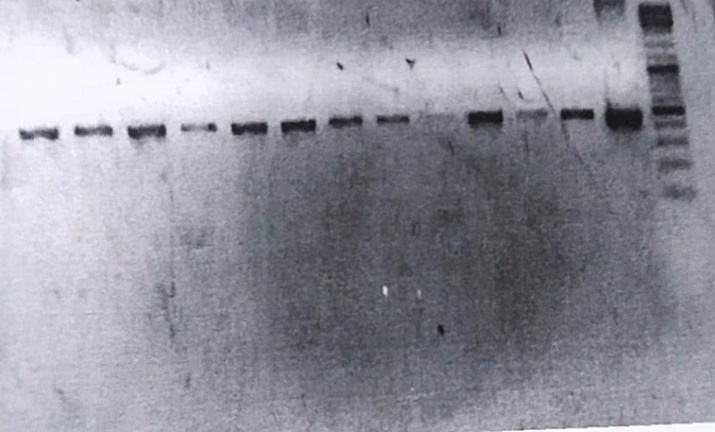


1000 bp

500 bp

**
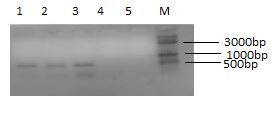
**

**Supplementary Fig. 4**. Results of PCR using deletion allele specific and wild-type specific allele specific primers in family RDHR07. **A)** Deletion specific primers against DNA of IV:2 (Lane 1), Control (Lane 2), III:10 (Lane 3), III:11 (Lane 4), detected mutant allele in all samples except that of a control. **B)** Deletion specific primers against DNA of III:10 (Lane 1), III:11 (Lane 2), IV:9 (Lane 3), IV:10 (Lane 4), V:5 (Lane 5), detected the mutant allele in carriers and patients. **C)** Deletion specific primers against DNA of III:10 (Lane 1), IV:10 (Lane 2), IV:4 (Lane 3), IV:5 (Lane 4), IV:6 (Lane 5), IV:11 (Lane 6), V:7 (Lane 7) confirmed absence of the mutant allele from multiple family members **D)** Wild-type allele specific primers against DNA of III:10 (Lane 1), III:11 (Lane 2), IV:2 (Lane 3), IV:4 (Lane 4), IV:5 (Lane 5), IV:6 (Lane 6), IV:11 (Lane 7), V:7 (Lane 8) detected the wild-type allele in these samples. **E)** Wild-type allele specific primers against DNA of III:10 (Lane 1), III:11 (Lane 2), IV: 2 (Lane 3), IV:9 (Lane 4), IV:10 (Lane 5). The wild-type allele is not amplified from the DNA of patients IV:9 and IV:10 confirming its absence from the genomic sequence. The last lane in all gel images has the DNA size standard ladder.

*
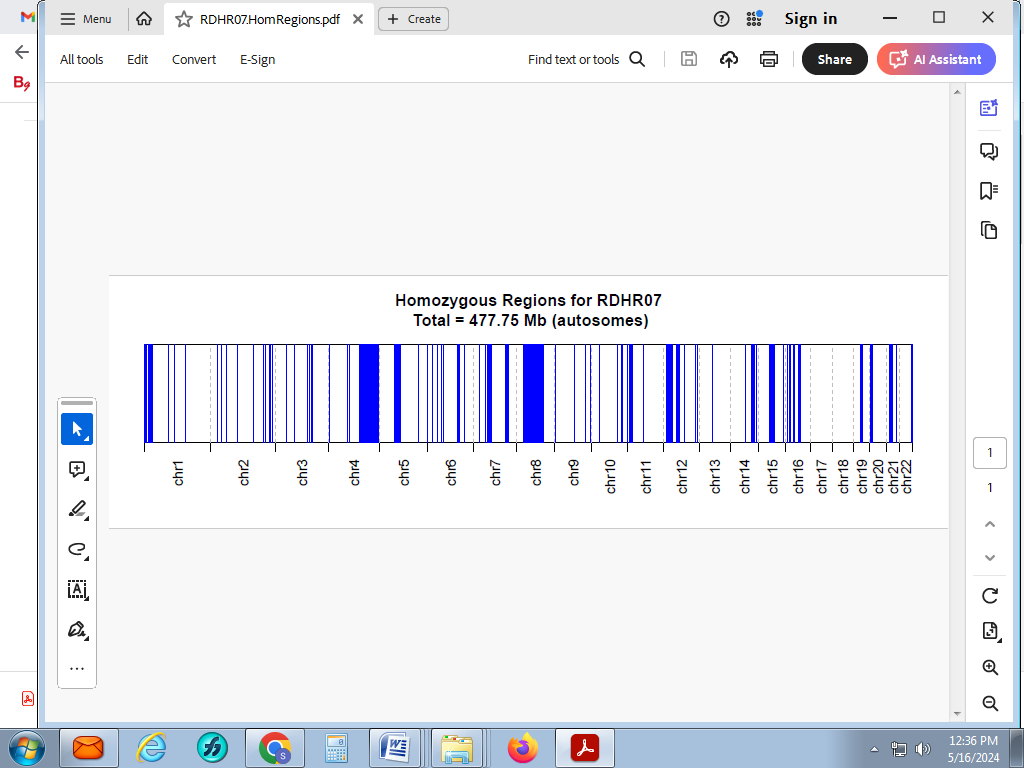
*


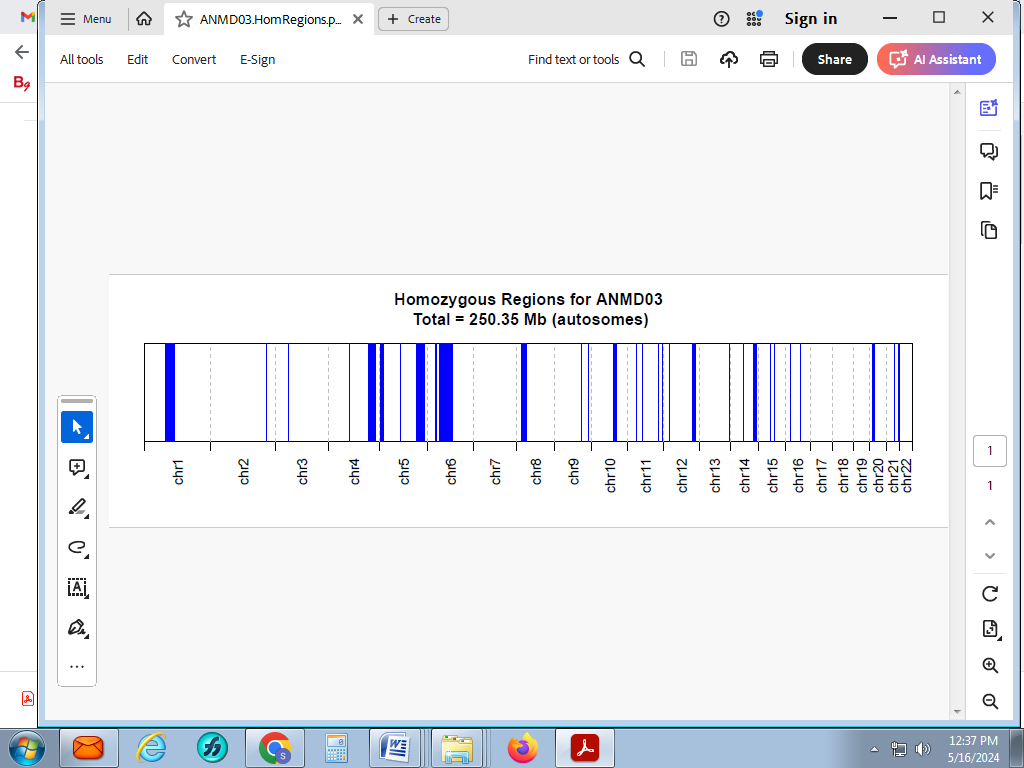


**Patient IV:10**

**Family RDHR07**

**Patient IV:3**

**Family ANMD03**

**Patient IV:3**

**Family RDFA06**


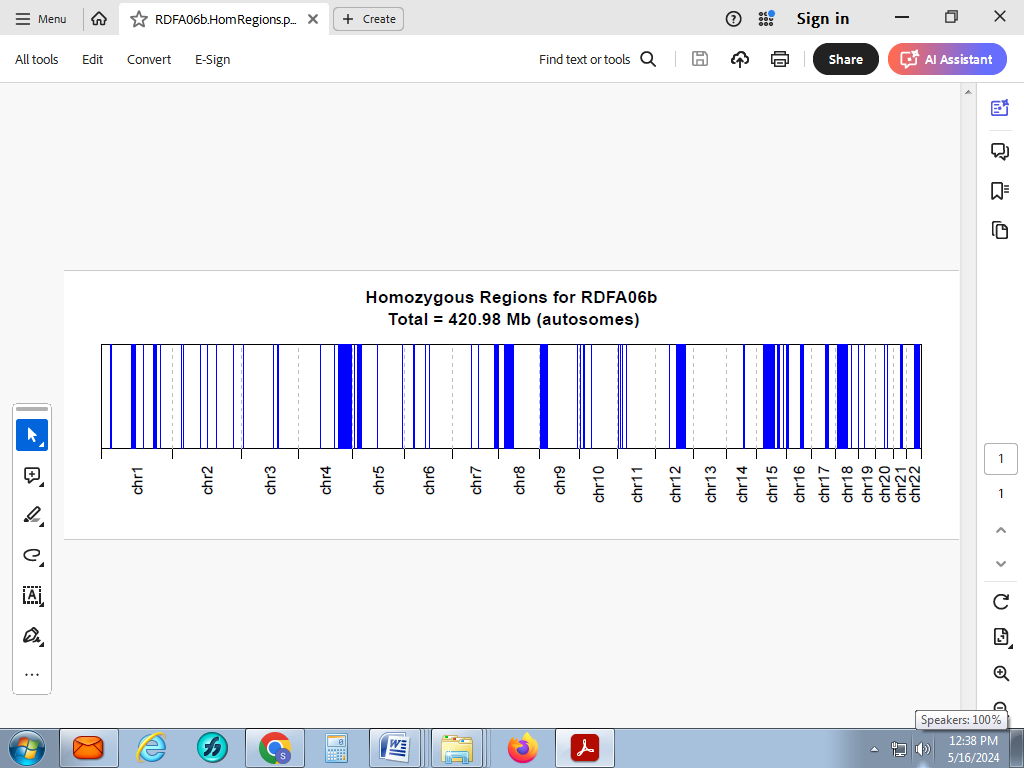


**Supplementary Fig. 5**: Display of autozygous regions by Automap for families RDHR07, ANMD03 and RDFA06 patients. The autozygous regions are visible as blue bars, starting from chromosome 1 and extending to the last autosome, chromosome 22. Note that all data have many autozygous regions which are of different sizes and are spread throughout the genomes. Some regions do not have ROH; for example, note chromosome 13 in the family RDFA06 patient data.

**A**


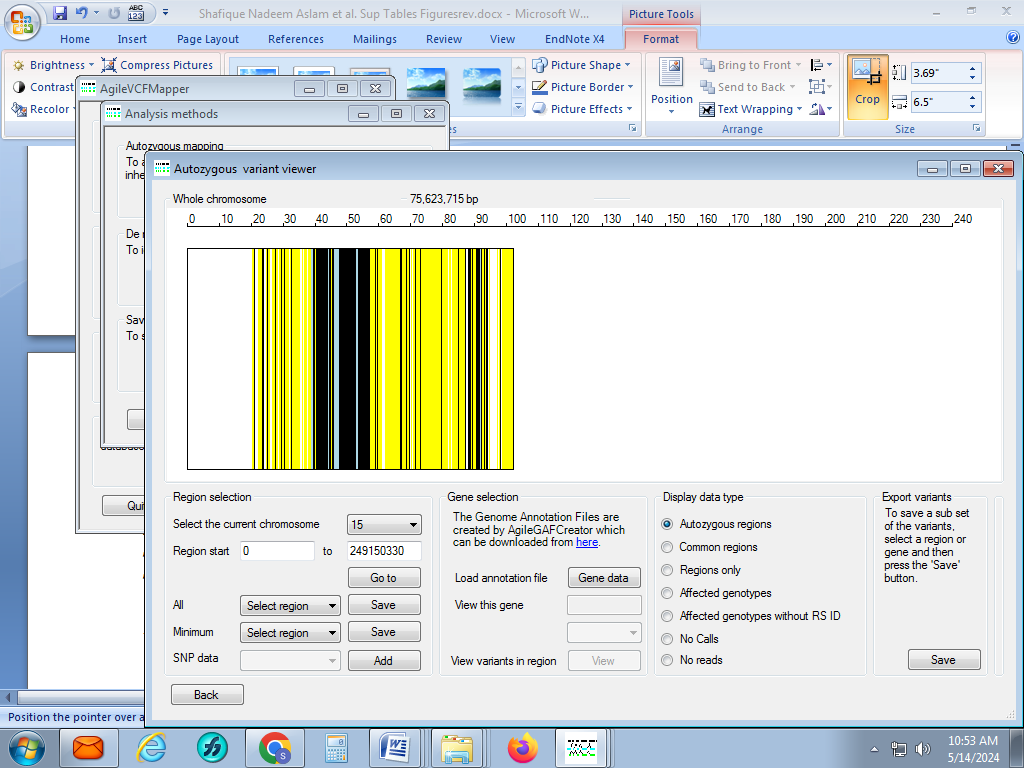


**Patient IV:10**

**Family RDHR07**

**Patient IV:3**

**Family ANMD03**

**Patient IV:3**

**Family RDFA06**

**ROH Chr15: ~38.7-57.8Mb, ~90.6-92.4 Mb**

**B**


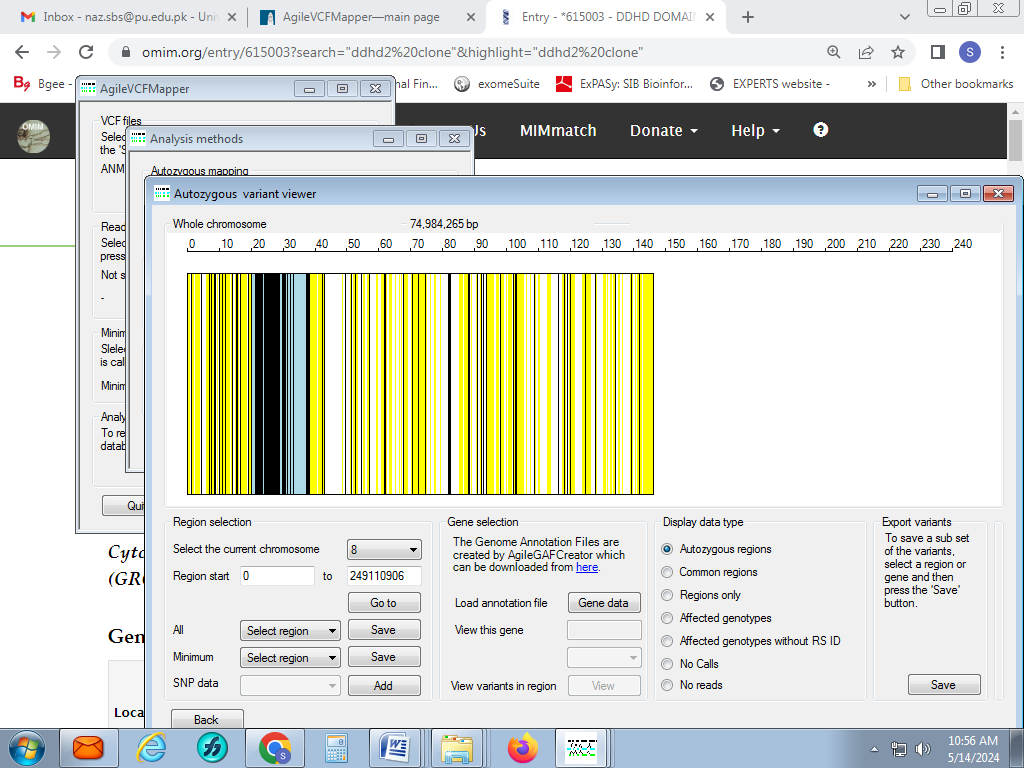


**ROH Chr8: ~19.9-38.44Mb**

**C**


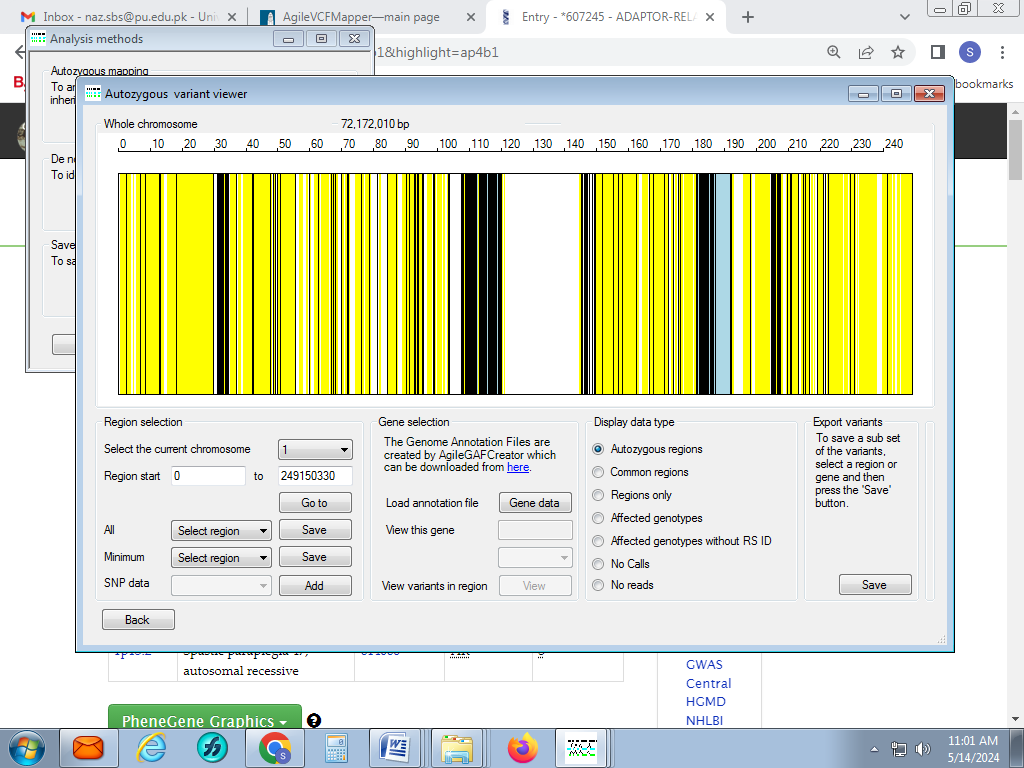


**ROH Chr1: ~30-34 Mb, ~106-120 Mb, ~145-150 Mb, ~180.8-190.6 Mb, ~204.7-209 Mb**

**Supplementary Fig. 6:** AgileVCFMapper selected displays of exome VCF data for the three participants. Each row corresponds to the mapped variant data of one patient for one chromosome starting from the telomere of “p” arm and ending at the telomere of the “q” arm. Yellow color/bars correspond to the heterozygous calls and the black/gray color/bars represent the homozygous calls. White denotes gaps due to the unsequenced centromeric sequences or detection of no variants in these regions. The chromosome number and the regions of homozygosity (ROH) are noted for each display. **A)** Chromosome 15 display for patient IV:10 in family RDHR07 had one major ROH spanning approximately 19 Mb and one ~2 Mb ROH. *SPG11* is present on chromosome 15 within the coordinates of the larger ROH. **B)** Family ANMD03 patient IV:3 data detected a ~18 Mb ROH on chromosome 8. The *DDHD2* gene is present within the coordinates of this interval. **C)** Family RDFA06 patient IV:3 data for chromosome 1. AgileVCFMapper detected five ROH on this chromosome with sizes ranging from ~4 to 14 Mb. The *AP4B1* gene is located at chr1:114,437,371-114,447,525 base pairs and is within the ~14 Mb ROH detected on chromosome 1.
